# Editorial

**Published:** 1865-03

**Authors:** 


### Editorial.

ANNUAL MEETING OF AMERICAN MEDICAL ASSOCIATION.—We are happy to learn from the Assistant-Secretary in Boston, that whatever differences of opinion had existed in the Committee of Arrangements, in regard to the Annual Meeting, have been reconciled; and that the Committee are now cordially united and actively preparing for the meeting on the first Tuesday in June next. We are assured that the members of the Association will meet with as cordial a reception in Boston, as has been extended to them in any other city; and we are confident that the business arrangements will be so perfect, as to render the meeting more interesting and profitable than any that have preceded it. Let every Medical Society in the North-West secure the appointment of such delegates as will attend.

---

CHICAGO MEDICAL SOCIETY—*Annual Meeting.*—The Annual Meeting of this Society was held on the evening of April 7th, 1865. The following officers and delegates were elected, viz.:—

For President,---------------- Thos. Bevan, M.D.

For Vice President,----------- E. Marguerat, M.D.

For Secretary and Treasurer, D. N. Tucker, M.D.

Delegates to the Illinois State Medical Society.—Drs. E. L. Holmes, John Reid, A. Fisher, O. Smith, and M. O. Heydock.

Delegates to the American Medical Association.—Drs. C. G. Smith, R. C. Hamill, G. Paoli, T. D. Fitch, and E. L. Holmes.

---

New York Medical Journal.—We have just received the first (April) number of a new medical journal, with the foregoing title, published in New York City. It is to be issued monthly, containing at least eighty pages, and the subscription price is *five* dollars per annum. The present number is published in excellent style, and its contents are varied and interesting. The only fault we have to find with it, is, the absence of the *name* of its editor. The names of a long list of able "collaborators" are given, but no editor, no responsible head, no master mind, whose individuality is to impress itself on the general character of the work. Notwithstanding this fault, however, we gladly place it on our exchange list, and cordially commend it to the patronage of the profession.

---

The Purification of the Press.—The London medical journals have entered upon an earnest crusade against the growing indecency of the public press in admitting to their columns the obscene advertisements of quacks, which promises the most satisfactory results. Already many of the leading papers throughout the kingdom have signified their intention to exclude such matter from their columns in future, and public opinion seems at last thoroughly aroused to the enormity of this offence against decency and morality. The immediate occasion of this healthy movement was the late exposure in the courts, by a victim of these extortionate quacks, who possessed a rare moral courage, of their nefarious system of robbing. Its success is all the more gratifying, as it suggests the possibility of similar reformation this side of the Atlantic. How much it is needed here need scarcely be told. We have but to look at the entire surface of some of our journals to find how largely they are devoted to the dissemination, not only of the grossest indecencies, but of future misery to a multitude of readers. Such papers are put into the hands of boys and girls of all ages, and find their way to the table of all classes of society, corrupting the purity of the young, arousing the fears of the weak, and encouraging vice by offers of immunity and relief. So outrageous has this system become, that no longer satisfied with the publicity of the largest type in our daily press, the venders of quack medicines and secret remedies paint their obscene notices in the most exposed places by every roadside. It is indeed time that our Legislature should turn its attention to this subject.

No one outside our profession, and we only to a small extent, can estimate the amount of bodily harm and loss of money inflicted upon the unwary and ignorant, who, induced by such advertisements, place themselves once within the power of these quacks. A large sum of money is at once demanded before the promised cure is undertaken, and the fears of the victim are so worked upon, during a treatment purposely designed to aggravate the disease, that he is obliged to pay additional sums until his means are exhausted. We were recently informed by a gentleman, whose official position led to the discovery, that two of the best houses in a certain fashionable street in this city were owned and occupied, under false names, by two of the most notorious of these advertising scoundrels.

There is another class of advertisements, less indecent, but quite as objectionable on the grounds of inhumanity and injustice to the people, of whom they profess to be the guardians, that fill the columns of our journals almost without exception. These are the notices of the innumerable remedies, patent medicines, and itinerants, which display their deceitful promises to the credulous eyes of the sick throughout the land. Our Sunday and religious papers are particularly devoted to the interests of these systems of robbery, and seem even less able to resist the temptation of a large fee than their more worldly daily brethren. The proprietors of such journals cannot screen themselves from the consequences of the course they pursue on the plea of an honest belief in the statements they publish, for no one is better informed than they of the short success of each new remedy or quack they introduce to the public, nor would they dare rely upon them for personal relief in sickness. The amount of evil they thus foster, and for which they are directly responsible, may be in part estimated by the magnitude of the sums received for advertising, which amount to millions in a single year. This, of course, represents only the pecuniary loss, which falls chiefly upon the poor and middle classes of society; the amount of physical suffering and disease thus occasioned cannot be estimated.

We hope that some of our leading journals will take this matter up, and not only proclaim their own intention of excluding all advertisements of this class in future, but, as in England, insist upon a uniform standard in this respect, upon which the respectability of all others shall be judged. Such a reform in their own conduct will give them the moral weight of self-purity in their judgment of the affairs of others, and gain the commendation and respect of all their readers.

The foregoing constitutes a recent editorial in the *Boston Medical and Surgical Journal.* We copy it for the purpose of fully endorsing its sentiments. There are but few things in modern society that illustrate more clearly the readiness with which a just sense of responsibility may be smothered for a pecuniary consideration, than the almost universal prostitution of the public press to the basest and most deceptive class of advertisements. Ask the proprietors of any respectable newspaper, whether secular or religious, if they have the slightest confidence in the pretensions set forth in the medical advertisements that help to fill its columns, and they will tell you promptly no; and freely admit that, as a general rule, they are simply lying and deceptive statements, designed to deceive the credulous and afflicted, for the sole purpose of bringing money to the pockets of their authors—in other words, a plausible mode of obtaining money under false pretences. And yet they attempt to screen themselves from all responsibility, under the plea that the statements are in the form of advertisements. However true such a plea may be in law, it certainly is not good in morals. For the confidence that a large proportion of newspaper readers place in the advertisements, is in direct ratio to the confidence they have in the reading matter of the paper generally, and in the intelligence of those who edit and publish it. Thus, you ask almost any one who has been spending his money for this or that *panacea*, why they were so foolish, and they will tell you promptly they saw it advertised and recommended in their own favorite newspaper, and they thought there must be some virtue in it, or it would not have been put into the *Tribune*, the *Times*, the New York *Independent*, or the *Christian Advocate*, as the case may be, thereby showing that the character of the paper has a direct influence on the confidence given to its advertisements. And there is no good reason why the editors and proprietors of a paper should not be held as rigidly responsible for the character of its advertisements, as for that of its editorials.

---

Sulphuric Ether versus Chloroform. By F. D. Lente, M.D., Cold Spring, N. Y.

Is it justifiable to use a remedy which has undeniably killed its hundreds, when we have an equally efficient one, with ordinary care, perfectly safe?

This is a question which should force itself upon the attention of every medical man who has occasion to use anæsthetics in surgery; it is a question of vital interest to his science and to his patients; it is a question which, I presume, any man would unhesitatingly answer in the negative; and yet a majority, perhaps, of surgeons are still risking the lives of their patients with chloroform, when they have, in sulphuric ether, an agent which has been *proved* to be equally efficacious, and which is admitted to be almost, if not altogether, perfectly safe. The question as to its safety, and the statistics bearing on the point, may be found fully discussed in an article furnished by me to the April (1861) number of the *American Journal of the Medical Sciences*, and in the Report of the Committee of the "Boston Society for Medical Improvement," published soon after, and need not be again canvassed now. As regards the proofs of its efficacy, it is only necessary to point to the hospitals in this country, but especially in Italy, where no other anæsthetic is used, and where immunity from pain is as perfect as elsewhere. But especially would I beg attention again to the statistics of the Mill Creek Military Hospital, Fortress Monroe, published by me in the June 28 number of the *American Medical Times* for 1862. There the quantity administered was reckoned by drachms, not by ounces, and the time one to two minutes. These results were witnessed, with considerable astonishment, by many military surgeons, and by some of the ablest civil surgeons and physicians in the country, some of them by the late Surgeon-General of the Army. Therefore their authenticity cannot be questioned. I have subsequently, also, in the same Journal, published, from my private practice, equally successful and not selected cases, duly authenticated by well known medical men. Therefore, this fact I regard as settled—that a patient may be brought under the influence of sulphuric ether as quickly as he can safely by chloroform, and with a quantity costing less, and weighing but little more than the requisite amount of the latter. The objection, then, sometimes raised by army surgeons, of increased trouble of transportation, is not tenable.

If any one doubts this fact, after referring to the statistics above alluded to, *I will agree to go to any hospital where a large number of operations are being performed, and demonstrate it to the satisfaction of the opponents of ether.* To be the humble means of preventing some of the sacrifice of life which is almost daily occurring from the use of chloroform in surgery, would warrant a much greater sacrifice of time and trouble than this.

The recently published report of the Royal Medical and Chirurgical Society of London, adverse to the employment of sulphuric ether, and the recent occurrence of so many additional cases of death from *chloroform poisoning*, have forcibly recalled my attention to this subject, and, at the risk of a charge of egotism, induced me to make the offer which I here repeat; in other words, that is, to guarantee to get a number of patients, in any hospital, under full anæthesia with sulphuric ether in as short a time as can safely be done with chloroform, and with a quantity not exceeding an average of two ounces and a half, the average time two minutes and a half. The average time and quantity would probably be less in the ordinary run of hospital cases, (not more than half as much in cases of considerable debility, and after hemorrhage or insufficient nourishment, as in many cases in military surgery.)

I close this article by quoting the "remarks" appended to a fatal case of chloroform poisoning, published in the last number of the *American Journal of Medical Science*, and by referring to another case of recent occurrence, not yet published in a medical journal, that I am aware of. "*Remarks.*—This case presents several points of interest. The drug was administered by a medical officer, five others being present, and it is unnecessary to say that due care was observed to guard against accident. Only a few weeks before, the anæsthetic had been given to the same individual in a much larger quantity, a fact which, in connection with the absence of visceral lesions, proves that there was nothing to contra-indicate the employment of the agent." These "remarks" are but a repetition of what we remark in every similar case, namely, that due care has been used, and that no human skill can enable us to foresee or prevent the dangers of chloroform. Can further comment be necessary? A former patient of mine, a young and gallant Colonel of one of our regiments, died recently in a neighboring village, of chloroform, administered, I believe, for the performance of some trifling operation.

The above is copied from the original department of the New York *Medical Journal*, for the purpose of fixing the attention of our readers, more fully, on the dangers attending the administration of chloroform, and of anesthetics generally. That the whole number of deaths induced by the use use of chloroform, directly, is very large, no one can deny. That many of these deaths have occurred without any fault in the time or mode of administering the agent, is equally certain; and, hence it cannot be truthfully said that its inhalation is actually free from danger in any single case. Its remote effects on the plasticity of the blood, and the properties of the solids, is also worthy of careful and thorough investigation. We strongly suspect that future investigations will show that the very general use of chloroform in surgical practice is far from having been an unalloyed boon to the human race. But, while we freely admit the dangers arising from the use of chloroform, is it true that sulphuric ether is actually *free* from danger, and can therefore be administered with impunity? That its inhalation is much less dangerous than chloroform, has been clearly shown by the statistics of the past. But that it is actually free from liability to produce fatal results, as would be inferred from the statements of Dr. LENTE, and others, we do not believe. Indeed, if ether could be at once substituted for chloroform, and thereby used by the same number and variety of patients, we do not believe ninety days would elapse without developing some fatal results. This opinion is founded partly on facts in relation to the effects of ether in several cases, in which it was administered by surgeens of much experience and skill, but in which respiration was so uniformly and completely suspended before sensibility was overcome, that the attempts at anesthesia had to be abandoned; and partly on the known relations of the different parts of the nervous system to each other. The practicability and safety of anesthesia, by any agent whatever, depend on the physiological facts that the nervous centres of ordinary sensation and voluntary motion are separate from those of respiration and circulation, and that the functions of the first are capable of being suspended by the inhalation of certain agents, without simultaneously suspending those of the latter also. The safety of the inhalation in any given case, depends on the order, as to time, in which the two sets of nervous centres are influenced by the agent used, and the skill of the operator in keeping the effect from extending beyond the centres controlling ordinary sensation and motion. Experience has shown that, in the best majority of individuals of both sexes, and all ages, chloroform, ether, and nitrous oxide, all affect the nervous centres of sensation, voluntary motion, and mental action, before materially influencing those of respiration, circulation, &c.; but it has equally shown that in a small percentage of the whole number, the reverse happens, and the excito-motory or involuntary centres are first influenced by the anesthetic. And this is, undoubtedly, the cause of most of the sudden and unexpectedly fatal results.

That chloroform is more apt to extend its influence to the excito-motory centres than ether, or nitrous oxide, and therefore much more dangerous, we have no doubt. But that the latter agents are absolutely safe, as represented by some, we do not believe. The whole subject of anesthetics and anesthesia, requires further investigation, in connection with the physiological relations of different portions of the nervous system to each other.

---

Illinois State Medical Society.—The following is the list of Committees expected to report at the Annual Meeting of the State Medical Society, to be held in Bloomington, on the fir Tuesday in May, 1865:—

*Committee on Practical Medicine and Epidemics:* J. Adams Allen, M.D., of Chicago; C. Goodbrake, M.D., of Clinton; H. Noble, M.D., of Hayworth.

*Committee on Surgery:* D. W. Young, M.D., of Aurora; A.

L. McArthur, M.D., of Joliet; L. T. Hewins, M.D., of Oak Alla.

*Committee on Obstetrics:* W. H. Byford, M.D., of Chicago; Lucius Clark, M.D., of Rockford; S. T. Trowbridge, M.D., of Decatur.

*Committee on Drugs and Medicines:* John Bartlett, M.D., of Chicago; F. R. Payne, M.D., of Marshall; T. D. Fitch, M.D., of Chicago.

*Committee on Ophthalmology:* E. L. Holmes, M.D., of Chicago; R. E. McVey, M.D., of Waverly; J. S. Whitmire, M.D., of Metamora; F. B. Haller, M.D., of Vandalia.

*Committee on Spotted Fever:* J. S. Jewell, M.D., of Chicago.

*Committee on Orthopœdic Surgery:* David Prince, M.D., of Jacksonville.

The Committee of Arrangements and the Profession in Bloomington are preparing to give the Society a cordial reception, and we trust the coming meeting will be larger and more profitable than any that have preceded it. We hope the committees will have their reports fully prepared.

---

American Medical Association.—The American Medical Association will hold its Sixteenth Annual Meeting in the City of Boston, on Tuesday, June 6th, 1865.

The following Committees are expected to report:—

On Insanity—Dr. H. R. Storer, Mass., Chairman.

On Exsection and its connection with Conservative Surgery—Dr. Lewis A. Sayres, N. Y., Chairman.

On Drainage and Sewerage of Large Cities, and their Influence on Public Health—Dr. W. J. C. Duhamel, D. C., Chairman.

On Alcohol and its Relations to Man—Dr. G. E. Morgan, Maryland, Chairman.

On Quarantine—Dr. Wilson Jewell, Penn., Chairman.

On Medical Ethics—Dr. John A. Murphy, Ohio, Chairman.

On the Microscope—Dr. James M. Corse, Penn., Chairman.

On the Relations which Electricity sustains to the Causes of Disease—Dr. Squire Littell, Penn., Chairman.

On the Morbid and Therapeutic Effects of Mental and Moral Influences—Dr. A. B. Palmer, Mich., Chairman.

On the Causes of the Extinction of the Aboriginal Races of America—Dr. Geo. V. Suckley, Md., Chairman.

On the Causes and Treatment of Ununited Fractures—Dr. Frank H. Hamilton, N. Y., Chairman.

On Diphtheria—Dr. Lucius Clark, Ill., Chairman.

On the Uses and Abuses of Pessaries—Dr. James P. White, N. Y., Chairman.

On International Medical Ethics—Dr. J. Baxter Upham, Mass., Chairman.

On Climatology and Epidemic Diseases—Dr. C. W. Parsons, R. I., Chairman.

On Autopsies in Relation to Medical Jurisprudence—Dr. T. C. Finnell, N. Y., Chairman.

On so-called Spotted Fever—Dr. James J. Levick, Penn., Chairman.

On the Introduction of Disease by Commerce, and the Means of its Prevention—Dr. A. Nelson Bell, N. Y., Chairman.

On Patent Rights and Medical Men—Dr. David Prince, Ill., Chairman.

On Revision of Plan of Organization—Dr. N. S. Davis, Ill., Chairman.

On Specialists—Dr. J. Homberger, N. Y., Chairman.

On Compulsory Vaccination—Dr. A. N. Bell, N. Y., Chairman.

On Rank of Medical Corps in the Army—Dr. C. S. Tripler, U.S.A., Chairman.

On Rank of Medical Corps in the Navy—Dr. T. L. Smith, N. Y., Chairman.

WM. B. ATKINSON, *Permanent Secretary.*

---

Practical Items.—The following paragraphs are copied from the proceedings of the Kings Co. Medical Society, as published in the *Buffalo Medical and Surgical Journal*, for March, 1865:—

*Specimen of Entozoa.*—Dr. Spier presented an interesting specimen of entozoa, taken from the right side of the heart of a dog. In the evening, the animal was in perfect health, but on the following morning was found dead. On opening the heart these *worms* were found (dead) in the right ventricle.

In reply to a question, the doctor stated that he had never found these animals in the human heart, but had discovered them in the kidneys. In this ventricle two were found, besides a clot of blood. The question here recurred, how did they get into the ventricle?

Dr. Enos looked upon it as a very interesting subject, and well worthy of being thoroughly sifted.

*Pyemia.*—Dr. Spier also presented a specimen of pyemia. The subject was a man who recently died in the Brooklyn City

Hospital. He had been operated upon a short time previously for a compound fracture of the leg. Gangrene set in, in the stump, and the man died. On *post mortem*, found the blood-vessels, especially the superficial veins, distended with air. The blood was also *oily*, which seemed under the microscope to be principally *margarine*. The liver was waxy, but the other organs of the body appeared to be healthy. Air was also found in the cephalic vein.

*Therapeutics*.—Dr. C. L. Mitchell called attention to his recent experience in the effects of certain remedies.

1.—Quinine, which for the last year he had found remarkably beneficial in allaying bronchial irritation, especially in children. He has used it in some cases of almost incessant cough, and had found it successful, after the failure of all the more usual anodyne expectorant remedies. In the bronchial irritation of pneumonia he had found it equally effectual and reliable. He cited the case of a child, aged 11, with excessive bronchial irritation, causing almost incessant cough, following measles. Skin dry, pulse 120, anorexia, and extreme feebleness. A grain of quinine every two hours, was followed by immediately beneficial effects, and a speedy recovery.

A case of hay asthma was last year much benefited, and this year wholly overcome by the use of quinine. He also alluded to the good effects of quinine in infantile convulsions, previously reported; and to its almost specific effects in neuralgia, especially in cases of miasmatic origin. He had recently had a most inveterate case, that had resisted other remedies, to whom he prescribed three grains every two or three hours, subsequently five grains, and finally ten every hour, which effectually subdued the disease, without any very unpleasant effects. There was a little tinnitus aurium, and decided sedative effects —lessened frequency of the pulse—and quiet sleep, of which the patient was in great need.

Dr. McClellan's experiences in the use of quinine, for the last year or two, confirmed that of Dr. Mitchell. He frequently prescribed it in bronchial irritation following measles or other causes, and considered it a remedy of great value in such cases. He had recently used it in two cases of children, with persistent barking cough, with perfect relief. In these cases he prescribed two grains four times a day.

*Turpentine in Chorea*.—Dr. Crane asked the experience of the members, in regard to the use of *turpentine in the treatment of chorea*.

Dr. McClellan related two cases which he had treated with turpentine, since hearing it spoken of by Dr. Crane. The first case was of two years' duration, in a child six years of age; turpentine was at first given in cathartic doses with marked benefit, and afterwards fifteen drops three times a day for a fortnight, when the chorea ceased. The second case came under his observation one month ago—was treated with small doses of turpentine with same result.

Dr. Crane reported a case of strongly marked chorea, a girl aged 10; this was an acute case. The patient was first treated with mercurial cathartics, and carb. ferri. She grew worse and worse, becoming idiotic, with loss of the power of locomotion and articulation, and the irregular motions persisted during sleep. He then gave two teaspoonfuls of turpentine on account of the mother's suspicion of worms. No worms were passed, but the patient's symptoms were relieved, and three days after the dose was repeated, with the same results. This was continued twice a week with marked benefit, for a month or six weeks, when the child was completely relieved, and there has been no recurrence of the disease.

*Removal of foreign bodies from the Nostrils.*—Dr. Enos exhibited a bean which was removed from the nose of a child about five years of age. He did so for the purpose of speaking of a simple mode of removing foreign bodies from the nostrils—a mode which, though not mentioned in the books, he has found very easy and efficacious. He would call it the blowing process. The manner is this: the child being in an upright position, place your mouth upon the *open* mouth of the child, and while with one finger you strongly compress the unobstructed nostril, blow somewhat forcibly and repeatedly, till the foreign body is forced out. The rationale of course is simple. When the air-passages of the child are filled, the column of air mounts over the velum into the nares, and impinges upon the posterior surface of the obstructing body, and forces it forward. Sometimes it will be forcibly ejected several yards.

---

THE LARYNX OF THE NEGRO. By GEORGE D. GIBB, M.D., &c. (Read before the meeting of the British Association for the Advancement of Science, September, 1864.)

The author's paper was upon the special difference between the larynx of the negro and the white man. After describing the larynx of the latter, he remarked that the essential point of difference between the two consisted in the invariable presence of the cartilages of Wrisberg, the oblique or shelving position of the true vocal cords, and the pendent position of the ventricles of Morgagni. Any one familiar, said the author, with the dissection or examination of the larynx in ourselves, cannot but perceive that these peculiarities are not observable, unless we will admit the occasional presence of the first in certain windpipes. Now, we may be told by some anatomists, that they have commonly seen these Wrisbergian bodies, and that they are not rare, but that sort of evidence counts for very little. These small bodies (the cartilages of Wrisberg) are either very minute and rudimentary, or wholly wanting in the white race, whilst they are large and well developed and always present in the black or colored races. It may be mentioned, also, that I have dissected them in monkeys, in whom, even the smallest species, they are relatively large in comparison to the size of their bodies; and, with the object of attracting attention to them in the quadrumana, I exhibited specimens before the Pathological Society of London, in March, 1861, three and a-half years ago. Those who argue that the black race are inferior to the white, and approach the quadrumana in some of their features, would naturally lay hold of what I have stated to prove the truth of this theory, especially as regards the Wrisbergian cartilages and the position of the ventricles. But I take the opportunity of declaring at once, that whatever views may be entertained by anthropologists respecting the position in the scale of being occupied by black and white, they are discarded from this communication.

Dr. Crisp remarked on this paper, that even if Dr. Gibb was right, and these cartilages pointed out existed, it was no proof of the degradation of the Negro. Extraordinary statements had been made on this subject. It might be that these particular cartilages were given to the Negro just the same as a black skin was given him, and thus did not imply the least degradation.

Mr. Carter Blake called attention to the statement of the late Prof. Eschricht, who found the cricothyroid muscles very large in the Negro, a portion of their fibres ascending to the internal surface of the thyroid cartilage. M. Pruner-Bey had suggested that this might be a trace of the internal cricothyroid muscles of the *hylobates*. Perhaps Dr. Gibb would offer some opinion on this hypothesis?

In reply to questions from the Rev. Dr. Macauley and Dr. Heaton, Dr. Gibb said that he had examined the larynxes of 100 white men and 58 blacks, and he had in every case found sufficient distinction to warrant him in introducing the subject to the notice of the Association.—*Anthropological Review, Nov.*, 1864, p. 322.

We commend this subject to the attention of American anatomists and anthropologists. In no other country in the world are there so many facilities for settling the point raised by Dr. Gibb as in our own, and we trust the matter will not be allowed to remain long in dispute.—*N. Y. Med. Jour.*

### REPORT OF THE WEIGHTS AND MEASURES USED IN PHARMACY.

By Mr. BARNARD S. PROCTOR.

[*Abstract.*]

"The author first made a comparison of the apothecaries' weights of our country with those of other civilized nations. Though there are forty different European pounds and as many ounces in general use, there were only two or three systems of pharmaceutical weights, and these not widely differing from each other. The English system, though good in the abstract, had no simple relation to the systems of other countries, nor to the other weights and measures of this country. Some of its own members were in an anomaulous position, What was a fluid pound of apothecaries' weight? Was it 12 avoirdupois ounces, 12 troy ounces, or 16 avoirdupois ounces? A critical examination was then made of several suggested alterations in the weights and measures of pharmacy, those of Mr. Jacob Bell, Mr. Griffin, Dr. C. Wilson, and Mr. Warington, being especially noticed. The advantages and disadvantages of the weights and measures authorized by the Medical Council in the new Pharmacopœia were next reviewed, and a suggestion made that the ounce of that system should be divided into drachms and scruples. To get rid of the fractions of a grain, which would otherwise be appended to these drachms and scruples, the author proposed that the value of the grain should be slightly increased; so that, instead of 18·229 grains being contained in one scruple, there should be only 18; instead of 54·687 in the drachm, there should be but 54; and 432 in the ounce, instead of, as now, 437·5. This was as near an approach to an amalgamation of the troy and apothecaries' system as he could devise. The elaborate and ambitious system proposed by the American Pharmaceutical Association was next noticed, and then the French metrical system, the merits and demerits of all under various circumstances being carefully weighed. For ultimate general adoption, the author thought the American octonary system to be superior to the metric decimal system; that, in short, doubling and halving a number was better than multiplying or dividing by ten. He concluded by proposing the use of the American system, modified to meet the requirements and customs of the English."—*Proc. Brit. Phar. Conf.*

---

THE BLACK TROOPS OF THE BRITISH ARMY AND CONSUMPTION.—There are some thousands of black troops in the service of the Crown. In Ceylon, the mortality is much lower among the native than among the white troops; but in the West Indies, where also there are both black and white, it is very decidedly otherwise. In Jamaica, the mortality among the black troops was 30.25 per 1000 of mean strength; among the white troops only 12.81. Mr. O'Flaherty, the principal medical officer in that command, remarks that the black soldier to outward view is apparently strong and muscular, but when sick, he has comparatively little power of resisting or sustaining disease, and fatal cases of consumption are seldom protracted to the advanced stages commonly observed among European soldiers. It must be borne in mind that the black recruit undergoes a very trying change, on enlisting, from almost complete idleness, and a semi-savage state of existence, to a life of order, regularity, and continued exertion in learning his work during the first two years; the white corps brings no soldiers in the recruit stage. In Jamaica, also, the black troops have much heavier duty than the white, and have been provided with only two meals a day, at 8 A.M., and at noon, leaving them for nearly twenty hours without any regularly provided sustenance: but the medical officer had recommended the addition of an evening meal. The liability of the black troops to consumption is remarkable, also, in the returns for West Africa. At the Gambia, the deaths from consumption and diseases of the lungs, in the four years, 1859–62, were as many as 17.64 per 1000 *per annum.* The mortality from all causes in the year 1862, exceeded 28 per 1000 at Sierra Leone, the Gold Coast, and Lagos; there are no European troops there to allow of a comparison of mortality.—*Brit. Med. Jour., Oct.* 29, 1865.

The increased experience of our own Army Medical Officers, has led them to opinions which are in accordance with those expressed in the foregoing article. The negro has not the ability to resist morbific influences which is possessed by the European, unless it is in the simple matter of malarious diseases, and even in this, there is room for doubt. The medical officers having charge of the negroes liberated by our armies on the lower Mississippi, unite in the opinion that they are not in the least exempt from the action of malarial poison, and the writer saw for himself that such is the case. Undoubtedly, however, a great deal of the sickness and mortality which prevailed among the blacks a year ago, in the localities referred to, was due to the entire neglect by those in power of the recommendations of the medical authorities, relative to food, clothing, and other matters affecting their hygienic condition.—*N. Y. Med. Jour.*

---

On the Internal Employment of Essence of Turpentine in the Headaches of Nervous Women.—M. Teissier thus describes the kind of cases of nervous headache in which he has found the essence of turpentine to be beneficial. The affection, he says, is a common, but often very severe one, and should not be confounded with ordinary neuralgia, either periodic or irregular, of the face or cranium, or even with hemicrania. This cephalæ is characterized by a much more fixed and continuous pain in the head, and may last not only several weeks, but months and entire years, without presenting more than rare and slight intermissions. The pain is sometimes dull, sometimes shooting, and sometimes pulsative, occupying only a single point of the head or the whole of the cranium, being accompanied by nausea or even vomiting, and complicated besides with much more serious symptoms, such as vertigo, and tendency to syncope, inability to think or to work, despondency, weariness of life, and, sometimes, numbness in the limbs. It is especially observed in nervous women, with exalted sensibility, of a delicate constitution, somewhat anæmic, and especially hysterical. It often co-exists with dysmenorrhœa, amenorrhœa, and also with a tendency to excessive menstruation, although it is sometimes observed in persons of good constitution whose menses are regular. M. Teissier observes that many remedies already exist which are efficacious in this complaint, such as valerian, assafœtida, the ethers, cyanide of potassium, aconite, &c.; and more particularly those which improve the blood, as chalybeate medicines, and different mineral waters. But these means sometimes fail, and then the essence of turpentine may be employed with advantage; although M. Teissier does not assert that it is infallible in its operation. It has been employed in the same kind of cases by Dr. Graves, and by Trousseau; but M. Teissier does not think it necessary to prescribe it in such large doses as those physicians have done. He recommends its use in capsules, given at meal-times, each capsule containing eight drops of the essence.—*Gazette Medical de Lyon, and Brit. & For. Med.-Chir. Rev.*

---

SUBSTITUTES FOR INDIAN INK.—A substance much of the same nature and applicable to the same purposes as Indian ink may be formed in the following manner:—Take of isinglass three ounces; make it into a size by dissolving over the fire in six ounces of soft water. Take then Spanish liquorice one ounce, dissolve it in two ounces of soft water over the fire in another vessel, then grind up on a slab with a heavy muller one ounce of ivory black with the Spanish liquorice mixture. Then add the same to the isinglass size while hot, and stir well together till thoroughly incorporated. Evaporate away the water, and then cast the remaining composition into a leaden mould slightly oiled, or make it up in any other convenient way. This composition will be found quite as good as the genuine article. The isinglass size mixed with the colors work well with the brush. The liquorice renders it easily dissolvable, on the rubbing up, with water, to which the isinglass alone would be somewhat reluctant; it also prevents it cracking and peeling off from the ground on which it is laid. A good Indian ink may be made from the fine soot from the flame of a lamp or candle received and collected by holding a plate over it. Mix this with the size of parchment, and it will be found to give a good deep color. Burnt rice has been by some considered a principal ingredient in the genuine Indian ink, with the addition of perfumes or other substances not essential to its qualities as an ink. —*Chemical News*, from *British Journal of Photography.*

---

PHYSIOLOGICAL EFFECTS OF DIGITALIS.—Dr. M. Galan presented to the Academy of Medicine in Havana, Cuba, a Memoir "on the Physiological Effects of Digitalis." From repeated experiments conducted by M. Martin-Magron, of Paris, Dr. Galan concludes that—

1st. Digitalis accelerates the pulse only transiently, and previous to the constant lessening which it produces on its action. 2d. This effect does not take place if the heart beats normally slow. 3d. Digitalis, like digitaline, as observed by Vulpian, causes a change in the shape of the heart. 4th. It diminishes the treating, both in organic and nervous affections of the heart. 5th. Hypertrophy is, of all affections of the heart, the most obstinate to the action of digitalis. 6th. This substance diminishes the calibre of the bloodvessels. 7th. It excites, instead of depressing, the cerebral functions. 8th. It very seldom determines convulsions attended with spinal paralysis. 9th. Digitaline may rationally be employed in several diseases: convulsions, epilepsy, spermatorrhœa, and specially in tetanus, in preference to woovara, which otherwise acts on the nerves, and excites the spinal cord, as proved by C. Bernard.—*Annals de la Real Academia de Ciencias Medicas, Fisicas y Naturales de la Habana. Dec.* 15, 1854.

---

MORTALITY OF CHICAGO for the month of March 1865, as compiled from the report of the Health Officer:—

DISEASES.

| | | | |
|---|---|---|---|
| Accidents and Injuries, | 7 | Old Age, | 9 |
| Bronchitis, | 5 | Paralysis, | 3 |
| Cancer of Stomach, | 3 | Pneumonia, | 27 |
| Childbirth, | 7 | Remittent Fever, | 1 |
| Cholera Infantum and Diarrhœa, | 8 | Rubeola (Measles), | 1 |
| Consumption, | 30 | Scarlet Fever, | 10 |
| Convulsions, | 27 | Scrofula, | 2 |
| Croup or Laryngitis, | 22 | Small-Pox, | 16 |
| Diphtheria, | 15 | Spotted Fever or Cerebro-Spinal Meningitis, | 5 |
| Disease of the Heart, | 5 | Stillborn, | 18 |
| Dropsy, | 6 | Typhoid and Typhus Fever, | 10 |
| Dysentery, | 2 | Unknown, | 9 |
| Erysipelas, | 3 | Whooping-Cough, | 2 |
| Inflammation of Brain, | 16 | | |
| Inflammation of Kidneys, | 1 | Total, | 279 |
| Inflammation of Liver, | 3 | | |
| Inflammation of Stomach & Bowels | 7 | | |

AGES OF THE DECEASED.—Under 5 years, 126; over 5 and under 10 years, 16; over 10 and under 20, 15; over 20 and under 30, 24; over 30 and under 40, 27; over 40 and under 50, 20; over 50 and under 60, 11; over 60 and under 70, 12; over 70 andunder 80, 4; over 80 and under 90, 3; stillborn, 18; unknown, 3. Total, 279.

The whole number of deaths for the same month, in the year 1864, was 345.

---

FOREIGN MEDICAL INTELLIGENCE.—Dr. Jonas Quain, author of Quain's Anatomy, died on the 31st of January, in London, at the age of 70 years.

There are 18,000 practitioners of medicine in France, at the present time.

We are sorry to announce the recent death of the eminent English geologist, Dr. Hugh Falconer. He has been engaged, for the last few years, in investigating the bone-deposits in caves.

Dr. Livingston is planning a new journey across the African continent.

The amount of absinthe drunk in Paris may be estimated in part by the fact that Switzerland alone sent 7,500,000 gallons thither last year.

The grand prize of 5000 francs has been adjudged, by the Académie des Sciences of Paris, to M. Roussell for his History of Pelagra; and, among others, one of 2500 francs to M. Zenker for his investigations on Trichiniasis. The grand prize in surgery, for 1866, of 20,000 francs, has for its subject, "The Preservation of the Limbs by the Preservation of the Periosteum."

---

DEMAND FOR PHYSICIANS.—The Medical Director of Philadelphia has received an order for twenty-six contract physicians to fill the vacancies in the Twenty-fifth Army Corps. If their services should prove satisfactory at the end of their three months' contract, they will be commissioned as assistant-surgeons without an examination. The new office of the Medical Director is at the N.E. corner of Broad and Spruce Streets.

